# Real-Time Changes in Corticospinal Excitability during Voluntary Contraction with Concurrent Electrical Stimulation

**DOI:** 10.1371/journal.pone.0046122

**Published:** 2012-09-26

**Authors:** Tomofumi Yamaguchi, Kenichi Sugawara, Satoshi Tanaka, Naoshin Yoshida, Kei Saito, Shigeo Tanabe, Yoshihiro Muraoka, Meigen Liu

**Affiliations:** 1 Department of Rehabilitation Medicine, Keio University School of Medicine, Tokyo, Japan; 2 The Japan Society for the Promotion of Science, Tokyo, Japan; 3 Division of Physical Therapy, Faculty of Health and Social Work, Kanagawa University of Human Services, Kanagawa, Japan; 4 Center for Fostering Young and Innovative Researchers, Nagoya Institute of Technology, Aichi, Japan; 5 Yokosuka Kyosai Hospital, Kanagawa, Japan; 6 Tokyo Bay Rehabilitation Hospital, Chiba, Japan; 7 Faculty of Rehabilitation School of Health Sciences, Fujita Health University, Aichi, Japan; 8 Faculty of Human Sciences, Waseda University, Saitama, Japan; University Medical Center Groningen UMCG, The Netherlands

## Abstract

While previous studies have assessed changes in corticospinal excitability *following* voluntary contraction coupled with electrical stimulation (ES), we sought to examine, for the first time in the field, *real-time* changes in corticospinal excitability. We monitored motor evoked potentials (MEPs) elicited by transcranial magnetic stimulation and recorded the MEPs using a mechanomyogram, which is less susceptible to electrical artifacts. We assessed the MEPs at each level of muscle contraction of wrist flexion (0%, 5%, or 20% of maximum voluntary contraction) during voluntary wrist flexion (flexor carpi radialis (FCR) voluntary contraction), either with or without simultaneous low-frequency (10 Hz) ES of the median nerve that innervates the FCR. The stimulus intensity corresponded to 1.2× perception threshold. In the FCR, voluntary contraction with median nerve stimulation significantly increased corticospinal excitability compared with FCR voluntary contraction without median nerve stimulation (*p*<0.01). In addition, corticospinal excitability was significantly modulated by the level of FCR voluntary contraction. In contrast, in the extensor carpi radialis (ECR), FCR voluntary contraction with median nerve stimulation significantly decreased corticospinal excitability compared with FCR voluntary contraction without median nerve stimulation (*p*<0.05). Thus, median nerve stimulation during FCR voluntary contraction induces reciprocal changes in cortical excitability in agonist and antagonist muscles. Finally we also showed that even mental imagery of FCR voluntary contraction with median nerve stimulation induced the same reciprocal changes in cortical excitability in agonist and antagonist muscles. Our results support the use of voluntary contraction coupled with ES in neurorehabilitation therapy for patients.

## Introduction

Cortical plasticity plays a fundamental role in motor learning and neurorehabilitation. Recently, voluntary contraction coupled with electrical stimulation (ES) has been reported to induce plasticity in the sensorimotor cortex in both health and disease [1]–[9]. These studies demonstrated that electrical stimulation combined with volitional efforts or additional therapy is behaviorally more effective than ES alone in the rehabilitation of patients following stroke [4]–[6]. In addition, Bhatt et al. [7] showed that this combined approach induced a more substantial cortical reorganization than either treatment alone. Thus, voluntary contraction coupled with ES appears to be a promising adjuvant therapy for functional recovery and brain reorganization after central nerve injury. However, the detailed effects of voluntary contraction coupled with ES on cortical plasticity remains largely unknown.

In previous studies, changes in cortical plasticity have been evaluated *after* the application of ES (offline effect or aftereffect) [1]–[3], [8]–[12]. For example, using transcranial magnetic stimulation (TMS) and the measurement of motor evoked potentials (MEPs), Khaslavskaia and Sinkjaer [3] demonstrated an increase in corticospinal excitability, an indicator of cortical plasticity, in stimulated muscle *after* voluntary contraction with ES. It is known that the after-effect of ES on cortical excitability is time dependent [1]–[3], [10]–[12]. However, the effect of ES *during* the very early period (i.e. in *real-time*) of intervention is entirely unknown. To gain a more *comprehensive understanding* of the mechanism underlying time-dependent effects of ES on cortical excitability, it is important to examine real-time effects, as well as offline effects, using real-time MEP measurement.

One technical difficulty encountered in recording real-time MEPs during ES is that ES perturbs to record the MEP signals using surface electromyography (EMG), generating electrical artifacts. In the present study, the mechanomyogram (MMG) was used instead to overcome this technical difficulty. The MMG monitors muscle vibrations and pressure waves generated by fused individual fiber twitches. Thus, the MMG is not affected by electrical artifacts. Reza et al. [13] and Hsieh et al. [14] demonstrated, using simultaneous EMG and MMG recordings, that the MMG could measure MEPs evoked by TMS as reliably as the EMG. In the present study, the MMG was used to measure MEPs evoked by TMS, and the differences in corticospinal excitability during voluntary contraction, with and without ES, were examined.

Using the MMG-MEP technique, we focused on the effects of voluntary muscle contraction combined with ES on the corticospinal excitability of agonist and antagonist muscle pairs, in real-time. Previous studies, while not examining real-time changes, suggested that (1) ES alone can induce facilitatory and inhibitory changes in corticospinal excitability in agonist and antagonist muscles, respectively (Kido Thompson and Stein [2]; but see also Knash et al. [12]). They have also suggested that (2) the effect of ES on corticospinal excitability is influenced by the cortical drive present during the ES [1], [3]. Thus, it has been hypothesized that (1) ES during voluntary muscle contraction induces real-time reciprocal facilitatory and inhibitory changes in corticospinal excitability in agonist and antagonist muscles, respectively; and that (2) these facilitatory and inhibitory changes are modulated by the strength of voluntary muscle contraction. In this study, low-frequency (10 Hz) ES was applied to the median nerve that innervates the flexor carpi radialis (FCR) during voluntary wrist flexion (FCR voluntary contraction). The stimulus intensity corresponded to 1.2× perception threshold.

Additionally, in another experiment we examined whether mental imagery of FCR voluntary contraction combined with median nerve stimulation would also change corticospinal excitability. This question is of particular importance when considering people with muscle weakness and low initial voluntary effort control in performing actual movements.

## Methods

Fifteen healthy right handed volunteers (8 males, 7 females), mean age of 30.6±5.4 years, with no history of neurological or psychiatric disease, participated in this study. All subjects provided written informed consent and the study was approved by The Ethical Committee of the Kanagawa University of Human Services and experiments were performed in accordance with the Helsinki Declaration.

The participants sat comfortably in a chair in front of a table, with the right forearm positioned horizontally over the table with the elbow flexed at an angle of 45° in the supinated position. ES was applied to the right median nerve that innervates the FCR during voluntary wrist flexion (FCR isometric voluntary contraction), using a pair of disposable surface electrodes (5 mm in diameter) at the elbow. The stimulus frequency was 10 Hz with a pulse duration of 1 ms [10], [11] and a hold time of 5 s. The stimulus intensity was the 1.2× perception threshold, and it did not induce muscle twitching or pain. The perceptual threshold is defined as the lowest stimulation that represents local activation of cutaneous receptors lying immediately underneath the stimulus electrodes [15]. Additionally, we carefully checked the intensity and voltage of median nerve stimulation on the monitor and asked the subjects whether they felt the changes of sensation elicited by median nerve stimulation during all experiments. The MEPs produced by TMS were recorded from both the FCR and the extensor carpi radialis (ECR) muscles during the following tasks: (i) at rest, i.e. 0% maximum voluntary contraction (MVC); (ii) 0% MVC with median nerve stimulation; (iii) 5% MVC; (iv) 5% MVC with median nerve stimulation; (v) 20% MVC; and (vi) 20% MVC with median nerve stimulation. All the participants performed the tasks in a random order. Voluntary contraction of wrist flexion was performed for the full 5 s with/without median nerve stimulation. We carefully checked the actual ECR EMG amplitudes during FCR contraction on the monitor. In addition, we asked participants to relax the ECR muscle when they contracted the ECR muscle. The MEPs were evoked at 4.5 s by TMS during each task, with an inter-stimulus interval of at least 10 s for each task.

The torque levels required during voluntary wrist flexion were at 5% and 20% of each subject's MVC. Torque levels were measured with a force transducer (Showa Measuring Instruments, Tokyo, Japan). The hand, in the intermediate position, with the fingers extended, was attached to the force transducer, and the subjects were required to press it with the tips of their four metacarpals excluding the thumb. The subjects were asked to maintain constant pressure on the force transducer at 5% or 20% of MVC during the tasks. The torque exerted was made visible as bar graphs on a computer screen placed in front of the subject.

TMS was performed using a figure-eight-shaped coil (diameter of the individual loop: 9 cm), connected to a Magstim 200 stimulator (Magstim, Whitland, UK), placed tangentially to the scalp in the optimal position (hotspot). The coil handle was held at an angle of 45° to the midsagittal line (approximately perpendicular to the central sulcus). The stimulus intensity was set at 120% above the resting motor threshold (RMT). The RMT was defined as the stimulus intensity that evoked reproducible MEPs of more than 50 µV five out of ten times in resting muscles.

The MMG response was detected by a contact square-shaped accelerometer element (Mechanomyogram Accelerometer, type MP101-10; Medisens, Sayama, Japan) on the FCR and ECR muscle bellies. The length and width of the accelerometer were 9 mm, and the thickness was 4 mm. The MMG signal was sent to an amplifier (Mechanomyogram MPS101; Medisens, Sayama, Japan) with a built-in band pass filter of 0.1–1 kHz. We recorded MEPs with the MMG during each task, and these were referred to as MMG-MEPs. The MMG-MEPs were elicited at each task 10 to 15 times. For each subject, the MMG-MEP response was measured as the peak to peak amplitude.

To investigate whether background muscle contraction levels affect the MMG-MEPs of both FCR and ECR muscles during each task, we calculated the background EMG of both FCR and ECR muscles in a 100 ms window before TMS by root mean square (RMS) analysis. One-way repeated measures ANOVA revealed a significant main effect of tasks (0% MVC; 0% MVC with median nerve stimulation; 5% MVC; 5% MVC with median nerve stimulation; 20% MVC; and 20% MVC with median nerve stimulation) on the RMS of the FCR muscle (F (5,70) = 67.70, p<.001). In contrast, there was no significant difference between voluntary contraction with or without median nerve stimulation in each voluntary contraction level. The RMS of the ECR muscle was not significantly different among the tasks (ANOVA, F (5,70) = 0.458, p = 0.806). Therefore, the background muscle contraction did not affect the results of MMG-MEPs during each task.

We investigated the effects of combined mental imagery of FCR muscle contraction with median nerve stimulation on corticospinal excitability in 7 subjects during the following tasks: (i) at rest, i.e. 0% MVC; (ii) 0% MVC with median nerve stimulation; (iii) mental imagery of 5% MVC; (iv) mental imagery of 5% MVC with median nerve stimulation; (v) mental imagery of 20% MVC; and (vi) mental imagery of 20% MVC with median nerve stimulation. The participants performed mental imagery of voluntary contraction after a training period of actual voluntary contraction (at each contraction level). We asked the participants to focus on bar graphs on a computer screen as well as tasks of actual voluntary contraction.

For each muscle, the effects of the combination of FCR volitional contraction with median nerve stimulation on the MMG-MEPs were analyzed using a two-way repeated measures ANOVA with factors of ES (with or without median nerve stimulation) and CONTRACTION (three levels of voluntary contraction; 0%, 5%, or 20% of MVC). For the effect of combined mental imagery of FCR muscle contraction with median nerve stimulation, we applied the same analysis to investigate whether the mental imagery of FCR muscle contraction combined with median nerve stimulation would also change the real-time corticospinal excitability. Post-hoc testing to detect significant differences for the various comparisons was performed using two-tailed multiple *t*-tests with a Bonferroni correction. Statistical analyses were performed using SPSS 15.0 for Windows. Statistical significance was defined as *p*<0.05 for all comparisons.

In addition, we also investigated whether the antagonist voluntary contraction effort with/without median nerve stimulation would change the real-time corticospinal excitability (for the methods and results, see supporting Text S1 and Figure S1).

## Results

**Figure 1:**
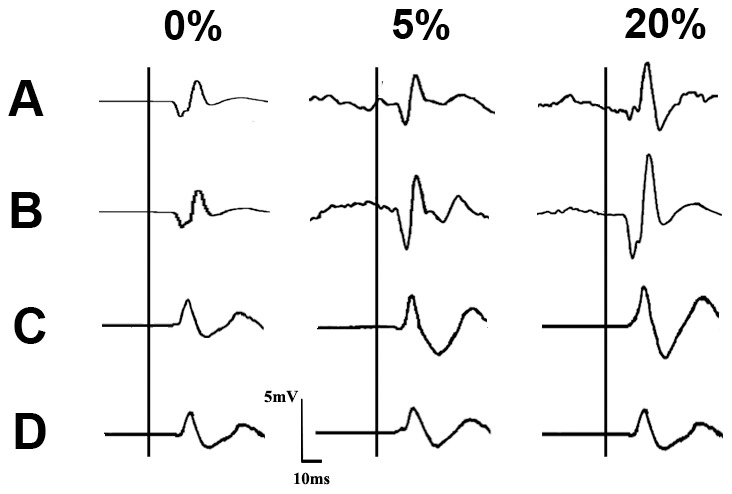
An example of MMG-MEPs during FCR voluntary contraction with and without median nerve stimulation in a single subject. The MMG-MEPs responses from FCR muscle during FCR voluntary contraction without median nerve stimulation (A) and with median nerve stimulation (B). (C) and (D) show the MMG-MEPs from ECR muscles during FCR voluntary contraction without median nerve stimulation and with median nerve stimulation, respectively. Left: Wave forms of MMG-MEPs during 0% maximum FCR voluntary contraction (at rest). Middle: Wave forms of MMG-MEPs during 5% of the MVC. Right: Wave forms of MMG-MEPs during 20% of the MVC. Black vertical lines show the trigger of TMS.

**Figure 2:**
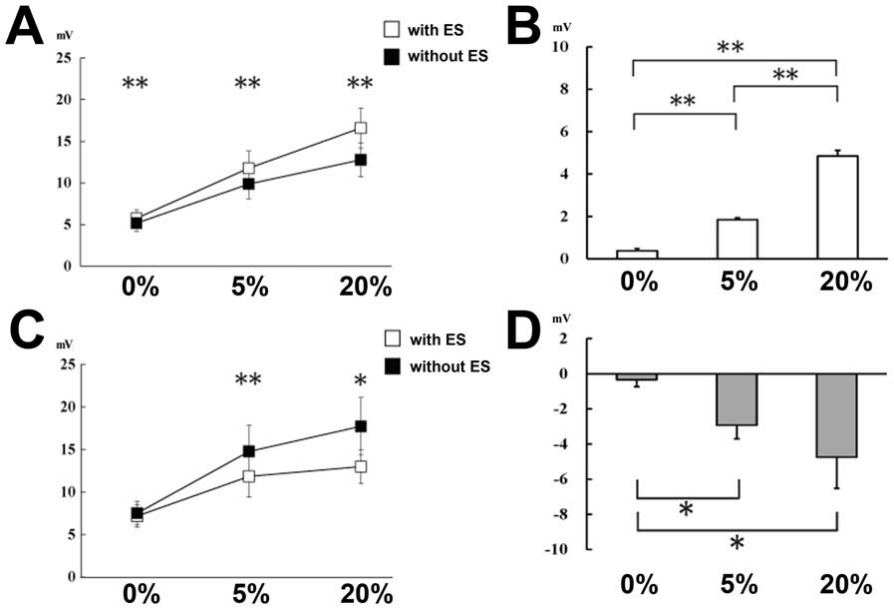
Changes in MMG-MEPs during rest or during FCR voluntary muscle contraction, with and without median nerve stimulation. Data are presented as the mean ± standard error (n = 15). Asterisks indicate significant differences between tasks by post-hoc testing with a Bonferroni correction (*p<0.05; **p<0.01). (A) MMG-MEPs from the FCR during 0, 5, and 20% of the FCR MVC with median nerve stimulation (open square) and without median nerve stimulation (filled square). (B) Effects of FCR voluntary contraction strength on corticospinal excitability measured in the FCR. The data from FCR voluntary contraction without median nerve stimulation was subtracted from the data from FCR voluntary contraction with median nerve stimulation. The FCR results are displayed as a white bar. (C) MMG-MEPs from the ECR during 0, 5, and 20% of the FCR MVC with median nerve stimulation (open square) and without median nerve stimulation (filled square). (D) Effects of FCR voluntary contraction strength on corticospinal excitability measured in the ECR. The data from FCR voluntary contraction without median nerve stimulation was subtracted from the data from FCR voluntary contraction with median nerve stimulation. The ECR results are displayed as a gray bar.

Figure 1 shows the row wave from change during FCR voluntary contraction with/without median nerve stimulation in FCR and ECR. The main FCR results are presented in Fig. 2A. A repeated measures ANOVA was performed with the two ES factors (with or without median nerve stimulation) and CONTRACTION (three levels of FCR voluntary muscle contraction; 0%, 5% or 20% of MVC). The analysis revealed significant main effects of ES (F (1,14) = 43.14, *p*<.001) and CONTRACTION (F (2,28) = 33.65, *p*<.001) as well as a significant interaction of ES and CONTRACTION (F (2,28) = 13.91, *p*<.001). The main effect of ES indicates that the MMG-MEP was higher when FCR voluntary contraction was coupled with median nerve stimulation than when it was not coupled with median nerve stimulation. The main effect of CONTRACTION indicates that the MMG-MEP amplitude increased with stronger muscle contraction. The significant interaction suggests that the effect of ES on the MMG-MEP was modulated by the level of FCR voluntary contraction. To test this possibility, a one-way repeated measures ANOVA with a CONTRACTION factor was applied to the delta MMG-MEP (the difference in MMG-MEPs between FCR voluntary contraction with and without median nerve stimulation, on each level of contraction; Fig. 2B). The analysis revealed a significant main effect (F (2,28) = 24.96, *p*<.001). Post-hoc analysis demonstrated that the delta MMG-MEP was significantly higher with 5% MVC than with 0% MVC (*p*<.001) and with 20% MVC than with 5% MVC (*p* = .003). These results indicate that median nerve stimulation during FCR voluntary contraction enhanced the MMG-MEP measured on the FCR (agonist muscle), and this facilitation increased with greater voluntary contraction.

In contrast, the application of median nerve stimulation during FCR voluntary contraction had an inverse effect on the MMG-MEP measured on the ECR (Fig. 2C). A repeated measures ANOVA revealed a significant main effect of ES (F (1,14) = 8.53, *p* = .012) and CONTRACTION (F(2,28) = 6.55, *p* = .005) and a significant interaction of ES and CONTRACTION (F (2,28) = 5.18, *p* = .012). The main effect of ES indicates that the MMG-MEP was lower when FCR voluntary contraction was combined with median nerve stimulation than when it was not combined with median nerve stimulation. The main effect of CONTRACTION indicates that the MMG-MEP amplitude rose with increasing strength of muscle contraction.

The one-way repeated measures ANOVA on the delta MMG-MEPs revealed a significant main effect (F (2,28) = 6.55, *p* = .005) in the antagonist muscle (ECR). Post-hoc analysis revealed that the delta MMG-MEP was significantly lower with 5% MVC compared with 0% MVC (*p* = .017), but there was no significant difference in the delta MMG-MEPs between the 5% MVC and the 20% MVC (*p* = .344). Therefore, these results indicate that the application of median nerve stimulation during FCR voluntary muscle contraction had an inhibitory effect on the MMG-MEPs measured on the ECR (antagonist muscle), and that the inhibitory effect became slightly stronger when subjects exerted a stronger drive (Fig. 2D).

**Figure 3:**
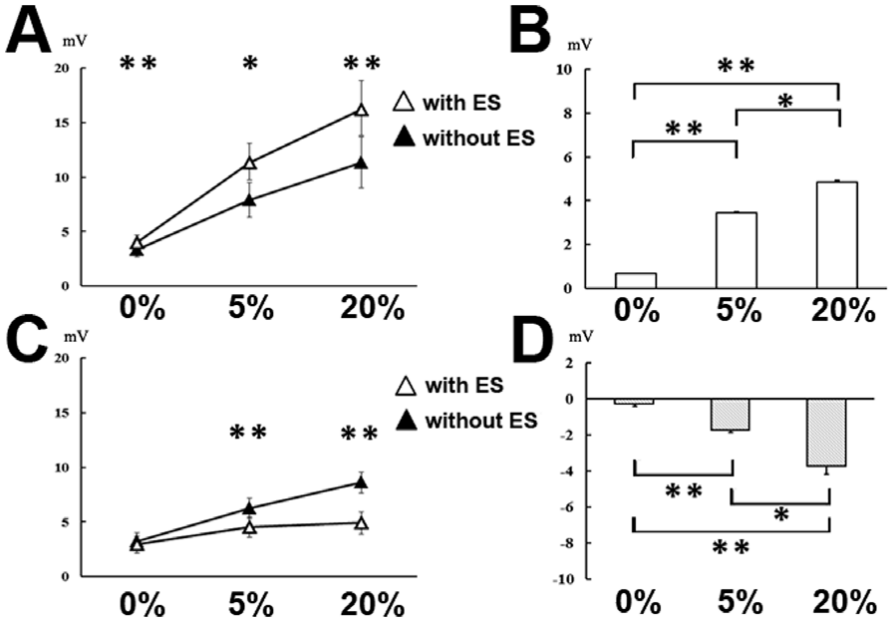
Changes in MMG-MEPs during rest or during mental imagery of FCR voluntary muscle contraction, with or without median nerve stimulation. Data are presented as the mean ± standard error (n = 7). Asterisks indicate significant differences between tasks after post-hoc testing with a Bonferroni correction (*p<0.05; **p<0.01). (A) MMG-MEPs from the FCR during mental imagery of 0, 5, and 20% of the MVC with median nerve stimulation (open triangle) or without median nerve stimulation (filled triangle). (B) Effects of mental imagery of FCR voluntary contraction strength on corticospinal excitability measured in the FCR. The data from mental imagery without median nerve stimulation were subtracted from those from mental imagery with median nerve stimulation. The FCR results are displayed as a white bar. (C) MMG-MEPs from the ECR during the mental imagery of 0, 5, and 20% of the MVC with median nerve stimulation (open triangle) or without median nerve stimulation (filled triangle). (D) Effects of mental imagery of FCR voluntary contraction strength on corticospinal excitability measured in the ECR. The data from mental imagery without median nerve stimulation were subtracted from data of mental imagery with median nerve stimulation. The ECR results are displayed as a gray bar.

Figure 3 shows the effects of combined mental imagery with median nerve stimulation. A two-way repeated measures ANOVA revealed a significant interaction of ES (with or without median nerve stimulation) and MENTAL IMAGERY (mental imagery of three levels of FCR voluntary contraction; 0%, 5%, or 20% of MVC) for the MMG-MEPs of FCR (F (2,12) = 9.85, *p* = .003) and ECR muscles (F (2,12) = 14.73, *p* = .001). Analysis of MMG-MEP amplitude in FCR revealed a significant main effect of ES (F (1,6) = 38.42, *p* = .001) and IMAGERY (F (2,12) = 13.06, *p* = .001). The main effect of ES indicates that the MMG-MEP was higher when mental imagery was combined with median nerve stimulation than when it was practiced alone (Fig. 3A). The main effect of IMAGERY indicates that the MMG-MEP amplitude increased with mental imagery of stronger voluntary contraction. To explore the difference between mental imagery of voluntary contraction strength with or without median nerve stimulation, a one-way repeated measures ANOVA with IMAGERY as the factor was applied to the delta MMG-MEP (Fig. 3B). The analysis revealed a significant main effect (F (2,12) = 47.85, *p*<.001). Post-hoc analysis demonstrated that the delta MMG-MEP was significantly higher with mental imagery of 5% MVC compared with mental imagery of 0% MVC (*p*<.001), and with mental imagery of 20% MVC compared with mental imagery of 5% MVC (*p* = .032).

Analysis of MMG-MEP amplitude in ECR revealed a significant main effect of ES (F (1,6) = 4.34, *p* = .002) and IMAGERY (F (2,12) = 19.09, *p*<.001). The main effect of ES indicates that the MMG-MEP was lower when mental imagery was combined with median nerve stimulation than without median nerve stimulation (Fig. 3C). The main effect of IMAGERY indicates the decrement of MMG-MEP amplitude with increasing mental imagery of voluntary contraction strength. For the delta MMG-MEPs in ECR, a one-way repeated measures ANOVA revealed a significant main effect (F (2,12) = 29.84, *p*<.001). Post-hoc analysis demonstrated that the delta MMG-MEP was significantly lower with mental imagery of 5% MVC compared with mental imagery of 0% MVC (*p* = .001) and with mental imagery of 20% MVC compared with mental imagery of 5% MVC (*p* = .033) (Fig. 3D).

## Discussion

In this study, we successfully used the MMG to measure MEPs during FCR voluntary muscle contraction with concurrent median nerve stimulation, in real-time. We made two major novel findings. First, FCR voluntary contraction combined with somatosensory afferent input by median nerve stimulation induces reciprocal facilitatory and inhibitory changes in corticospinal excitability of agonist and antagonist muscles, respectively. Second, these facilitatory and inhibitory changes are modulated by the strength of FCR voluntary muscle contraction. These results have supported our hypothesis.

The combination of voluntary training and ES has been reported to produce long-lasting changes in the excitability of the motor cortex (although hitherto not measured in real-time) responsible for controlling the tibialis anterior [2], [3]. The effects were greater than with either electrical stimulation or voluntary movement alone. We consider that a combination of voluntary contraction and somatosensory input by electrical stimulation may lead to a summation effect in the motor cortex for the control of the agonist muscle. Thus, these effects might effectively strengthen corticospinal circuitry. The temporal features of the synaptic input to neurons might be a crucial factor for the induction of plasticity [16]. In contrast, the corticospinal excitability of the antagonist muscle showed significant decreases during FCR voluntary contraction coupled with median nerve stimulation in the present study. This result is different from that of Kido Thompson and Stein [2], in which the corticospinal excitability of the antagonist muscle increased after 30 min of walking in combination with ES to the common peroneal nerve. The reason for this discrepancy is unknown, although it is possible that differences in the effector (the upper limb in the present study, in contrast to the lower limb in their study) and in the skillfulness of movement, as well as in the timing of the MEP measurements (real-time in the present study; offline in their study), might be responsible. The effect of electrical nerve stimulation on corticospinal excitability is dependent on the stimulus parameters (e.g., stimulus intensity, frequency, and pulse duration) [17]. The effects of electrical stimulation on corticospinal excitability during voluntary contraction might be different when the stimulus parameters are changed.

Our results demonstrate that the difference between with and without median nerve stimulation in amplitude of the MMG-MEPs in the FCR increased with each strength of FCR muscle contraction, despite the fact that the sensory input provided by median nerve stimulation was the same for all levels of muscle contraction. These results suggest that the summation of combined voluntary contraction and somatosensory input may be modulated by dynamic changes in the presence of voluntary drives. During voluntary muscle contraction, the degree of excitability in the primary motor cortex is modulated, possibly due to dynamic changes in voluntary drive [18]–[20] and afferent input [21]. Indeed, changes in the excitability of the motor cortex appear to be dependent not only on the extent of voluntary drive for contracting the agonist muscle, but also on afferent input provided by muscle contraction [22], [23].

We observed that median nerve stimulation during FCR voluntary muscle contraction induces reciprocal facilitatory and inhibitory changes in MMG-MEPs in agonist and antagonist muscles, respectively. We speculate that input from muscle afferents during FCR voluntary contraction with median nerve stimulation can induce transient facilitation in the corticospinal tract that control the agonist muscle (FCR) and transient inhibition in neurons that project to the corticospinal tract of the antagonist muscle (ECR). Mechanistically, the basal ganglia may modulate cortical activation by filtering and focusing thalamocortical input. A conceptual model suggests that the basal ganglia facilitate specific motor pathways and inhibit competing motor pathways by modulating the facilitation and inhibition of their cortical target outputs [24]–[26]. The results of Bertolasi et al. [27] and Rosenkranz et al. [28] suggest that afferent input from the forearm flexor muscles activates a reciprocal connection between agonist and antagonist muscles within the motor cortex. Alternatively, changes in the excitability of spinal interneurons might contribute to the results observed in the present study [29]. Furthermore, the excitability of spinal interneurons might be accompanied by an increase in the excitability of the MMG-MEPs in the FCR, most likely due to an increase in descending volleys reaching spinal interneurons. On this point, the excitability of motoneurons and spinal interneurons could not be measured in the present study because the median nerve was electrically stimulated during tasks, and it was technically difficult to evoke an H-reflex simultaneously.

In the present study, median nerve stimulation alone had a significant effect on MEP size in the FCR. This finding is consistent with previous studies [1]–[3], [10]–[12]. Functional magnetic resonance imaging (fMRI) studies [30]–[32] indicate that ES modulates not only activity in the primary motor cortex, but also activity in multiple brain areas including the somatosensory cortex. Thus, it is possible that the combination of voluntary contraction and ES may increase brain activity in other brain regions in addition to the primary motor cortex in the present study. Future studies should examine activity in other brain regions after the combination of voluntary contraction and ES using fMRI.

MMG-MEPs recordings by TMS have been described in humans and shown to be highly correlated with the EMG-MEPs [13]. Hsieh et al. [14] also showed that the TMS-MMG rodent embodiment of human TMS protocols also could evaluate the cortical excitation and inhibition in rats. With increasing TMS intensity, MMG amplitudes increased in proportion to the machine output to produce reliable input-output curves. Therefore, MMG-MEPs could provide reliable information of corticospinal excitability. The EMG cannot precisely record MEPs due to electrical artifacts induced by concurrent ES. In the present study, we could measure MMG-MEPs during median nerve stimulation without electrical artifacts in real-time. The MMG is an adequate substitute for EMG to record corticospinal excitability with TMS during concurrent ES.

Together with previous findings [1]–[9], [33], our results again suggest that the combination of voluntary contraction and sensory electrical stimulation might be an adjuvant therapy for motor rehabilitation, because sensory electrical stimulation does not disturb active movement significantly. For example, it is possible to use this method for locomotor training such as treadmill walking with partial body weight support [34], robot-assisted locomotor training [35], or pedaling exercise [36]. In addition, the present study also indicated that corticospinal excitability is modulated by the level of voluntary contraction. This means that active movement by daily activities and training [9], [33] may selectively increase corticospinal excitability. These aspects, together with the proven efficacy of the combination therapy on functional recovery and brain reorganization in patients with central nervous system injuries [4]–[9], suggest that the combination of voluntary contraction and ES can be effectively used in rehabilitation programs for these patients.

In conclusion, our study suggests that the combination of FCR voluntary contraction and sensory afferent input by median nerve stimulation can induce nearly instantaneous changes in corticospinal excitability. Combination of mental imagery with median nerve stimulation also induced the same effects on corticospinal excitability. This finding might be useful for treatment of people with muscle weakness and low initial voluntary effort control. Our results support the notion that a combination of voluntary cortical drive and ES induces use-dependent plasticity and reorganization in central motor structures. Thus, voluntary contraction coupled with ES might be useful in neurorehabilitation therapy for injuries to the central motor system.

## Supporting Information

Text S1 **Real-time changes in corticospinal excitability during the antagonist voluntary contraction effort with/without median nerve stimulation.**
(DOC)

Figure S1 **Changes in MMG-MEPs during rest or during antagonist voluntary contraction (ECR voluntary contraction), with and without median nerve stimulation.**
(TIF)
