# Supplementary material for: Real-Time Changes in Corticospinal Excitability during Voluntary Contraction with Concurrent Electrical Stimulation
Source: PLoS One. 2012 Sep 26;7(9):e46122. doi: 10.1371/journal.pone.0046122 (PMC3458815; doi:10.1371/journal.pone.0046122)
Supplement: Text S1 — Real-time changes in corticospinal excitability during the antagonist voluntary contraction effort with/without median nerve stimulation. (DOC) [file pone.0046122.s001.doc]

Real-time changes in corticospinal excitability during voluntary contraction with concurrent electrical stimulation

Tomofumi Yamaguchi, Kenichi Sugawara, Satoshi Tanaka, Naoshin Yoshida, Kei Saito, Shigeo Tanabe, Yoshihiro Muraoka, Meigen Liu

**Supporting information legends**

Text S1

Method

We investigated whether the antagonist voluntary contraction (ECR voluntary contraction) effort with/without median nerve stimulation would change the real-time corticospinal excitability in 7 subjects during the following tasks: (i) at rest, i.e. 0% maximum voluntary contraction (MVC); (ii) 0% MVC with median nerve stimulation; (iii) antagonist voluntary contraction of 5% MVC; (iv) antagonist voluntary contraction of 5% MVC with median nerve stimulation; (v) antagonist voluntary contraction of 20% MVC; and (vi) antagonist voluntary contraction of 20% MVC with median nerve stimulation.

Result

A two-way repeated measures ANOVA revealed that there was no significant interaction of ES (with or without median nerve stimulation) and CONTRACTION (three levels of ECR voluntary contraction; 0%, 5%, or 20% of MVC) for the MMG-MEPs of FCR (F (2,12) = 1.27, *p* = .315) and ECR muscles (F (2,12) = 0.89, *p* = .436). Analysis of MMG-MEP amplitude in FCR revealed a significant main effect of CONTRACTION (F (2,12) = 5.52, *p* = .020) (Fig. S1A). There was no main effect of ES (F (1,6) = 0.91, *p* = .376). Analysis of MMG-MEP amplitude in ECR revealed a significant main effect of CONTRACTION (F (2,12) = 4.23, *p* = .041) (Fig. S1B). There was no main effect of ES (F (1,6) = 0.54, *p* = .491). The main effect of CONTRACTION indicates the decrement of MMG-MEP amplitude with increasing antagonist voluntary contraction strength. Therefore, at least in this experimental setting, median nerve stimulation did not significantly affect corticospinal excitability during antagonist voluntary effort. The reason for this negative result is unclear.

Figure S1

Figure S1. Changes in MMG-MEPs during rest or during antagonist voluntary contraction (ECR voluntary contraction), with and without median nerve stimulation.

Data are presented as the mean ± standard error (n = 7). (A) MMG-MEPs from the FCR during the antagonist voluntary contraction (ECR voluntary contraction) of 0, 5, and 20% of the MVC with median nerve stimulation (open diamond) or without median nerve stimulation (filled diamond). (B) MMG-MEPs from the ECR during the ECR voluntary contraction of 0, 5, and 20% of the MVC with median nerve stimulation (open diamond) or without median nerve stimulation (filled diamond).
