# Supplementary figures and images for: Real-Time Changes in Corticospinal Excitability during Voluntary Contraction with Concurrent Electrical Stimulation
Source: PLoS One. 2012 Sep 26;7(9):e46122. doi: 10.1371/journal.pone.0046122 (PMC3458815; doi:10.1371/journal.pone.0046122)

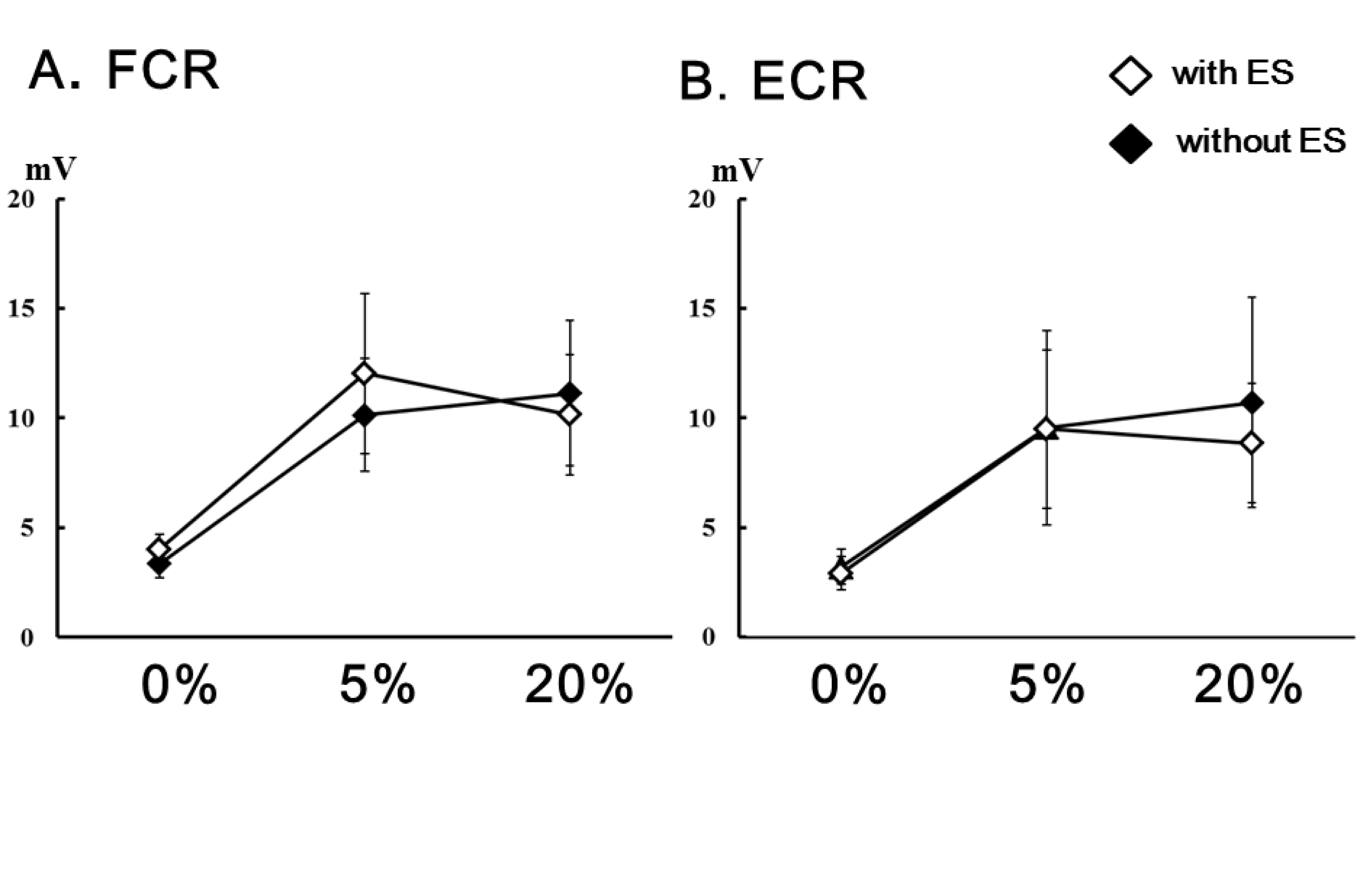

Supplement: Figure S1 — Changes in MMG-MEPs during rest or during antagonist voluntary contraction (ECR voluntary contraction), with and without median nerve stimulation. (TIF) [file pone.0046122.s002.tif]
